# Classification-Based Inference of Dynamical Models of Gene Regulatory Networks

**DOI:** 10.1534/g3.119.400603

**Published:** 2019-10-16

**Authors:** David A. Fehr, Joanna E. Handzlik, Manu, Yen Lee Loh

**Affiliations:** * Department of Physics and Astrophysics, and; † Department of Biology, University of North Dakota, Grand Forks, ND 58202

**Keywords:** drosophila, gap gene regulation, differential equation models, pattern formation, parameter inference, binary classification, development, gene regulatory networks

## Abstract

Cell-fate decisions during development are controlled by densely interconnected gene regulatory networks (GRNs) consisting of many genes. Inferring and predictively modeling these GRNs is crucial for understanding development and other physiological processes. Gene circuits, coupled differential equations that represent gene product synthesis with a switch-like function, provide a biologically realistic framework for modeling the time evolution of gene expression. However, their use has been limited to smaller networks due to the computational expense of inferring model parameters from gene expression data using global non-linear optimization. Here we show that the switch-like nature of gene regulation can be exploited to break the gene circuit inference problem into two simpler optimization problems that are amenable to computationally efficient supervised learning techniques. We present FIGR (Fast Inference of Gene Regulation), a novel classification-based inference approach to determining gene circuit parameters. We demonstrate FIGR’s effectiveness on synthetic data generated from random gene circuits of up to 50 genes as well as experimental data from the gap gene system of *Drosophila melanogaster*, a benchmark for inferring dynamical GRN models. FIGR is faster than global non-linear optimization by a factor of 600 and its computational complexity scales much better with GRN size. On a practical level, FIGR can accurately infer the biologically realistic gap gene network in under a minute on desktop-class hardware instead of requiring hours of parallel computing. We anticipate that FIGR would enable the inference of much larger biologically realistic GRNs than was possible before.

Development is controlled by gene regulatory networks (GRNs) that integrate extrinsic signals and intrinsic cell state to make decisions about cell fate (Levine and Davidson 2005; Davidson and Levine 2008). Modeling of GRNs is an important approach to understanding a wide variety of developmental processes such as pattern formation (Manu *et al.* 2009a,B; Verd *et al.* 2018; Balaskas *et al.* 2012), cell-fate specification (Hamey *et al.* 2017; May *et al.* 2013), pluripotency and cell-fate reprogramming (Collombet *et al.* 2017; Li and Wang 2013), oncogenesis (Tyson *et al.* 2011), and regeneration (Pietak *et al.* 2019). Over the past decade or so, it has become clear that developmental GRNs comprise tens to hundreds of densely interconnected genes (Davidson *et al.* 2002a; Novershtern *et al.* 2011) rather than a few so-called master regulators. Moreover, developmental GRNs are wired recursively since the genes encoding transcription factors (TFs) are themselves regulated by other TFs or indirectly by non-TF gene products (Palani and Sarkar 2008; Kueh *et al.* 2013). Their large size and high interconnectivity make the modeling of developmental GRNs a challenging problem.

Coupled ordinary or partial differential equations (ODEs or PDEs) are a natural choice for modeling GRNs since GRNs are nonlinear dynamical systems (Manu *et al.* 2009a; Weston *et al.* 2018; Li and Wang 2013; Laslo *et al.* 2006) whose time evolution depends on their state. The state is defined by the concentrations of gene products and the equations are parameterized by constants with a biochemical or biophysical underpinning, such as synthesis and degradation rates and binding constants. Estimating the values of these parameters is necessary for simulating the time evolution of GRN state but direct *in vivo* biochemical measurement of the large numbers of parameters involved is generally infeasible if not outright impossible. One approach to estimating parameter values is to search in parameter space for broad regions that reproduce the qualitative behavior of the system (Huang *et al.* 2007; Laslo *et al.* 2006; Li and Wang 2013; Hong *et al.* 2012). The other approach (Reinitz and Sharp 1995; May *et al.* 2013) to parameter estimation is data-driven, that is, parameter values are inferred by fitting the ODEs or PDEs to quantitative observations of GRN state sampled in space and/or time. In inferring parameters from quantitative data, data-driven differential equation modeling of GRNs provides a framework for understanding developmental cellular decisions at a quantitative and predictive level.

Here we focus on a specific data-driven and predictive ODE modeling framework, termed *gene circuits*, that has been particularly successful in inferring and modeling developmental GRNs from spatiotemporal protein (Jaeger *et al.* 2004a; Manu *et al.* 2009a,b; Kozlov *et al.* 2012; Hengenius *et al.* 2011) or mRNA (Crombach *et al.* 2012) data. Gene circuits determine the time evolution of protein or mRNA concentrations using coupled nonlinear ODEs in which synthesis is represented as a switch-like function of regulator concentrations. The values of the free parameters define the regulatory influences among the genes in the network. Gene circuits do not presuppose any particular scheme of regulatory interactions, but instead determine it by estimating the values of the parameters from quantitative data using optimization. Gene circuits infer not only the topology of the GRN but also the type, either activation or repression, and strength of interactions. Most importantly, the inference procedure yields ODE models that can be used to simulate and predict developmental perturbations (Jaeger *et al.* 2004b; Manu *et al.* 2009a,b; Wu *et al.* 2015; Verd *et al.* 2018).

Despite its successes, the gene circuit method suffers from the drawback that parameter inference is computationally expensive. Efficient optimization methods, such as steepest descent (Gursky *et al.* 2004) are guaranteed to find the global minimum only if the cost function, usually the sum of squared differences between model output and data, is convex—has a unique minimum—which is not the case in such problems. This implies that the only practical approach currently available for fitting gene circuit models is global nonlinear optimization with techniques such as simulated annealing (SA; Kirkpatrick *et al.* 1983; Lam and Delosme 1988a, b), that minimize the cost function by searching the high-dimensional parameter space stochastically. Not only do global nonlinear optimization methods need to make millions of cost function evaluations in order to find the minimum, but each evaluation is itself quite costly since it involves solving a set of coupled differential equations. Furthermore, the computational cost scales poorly, as $O\left(G^{3}\right)$, with gene number *G*, since *G* ODEs are solved in each function evaluation and the number of cost function evaluations required is proportional to the number of parameters ($O\left(G^{2}\right)$). High computational cost and poor scalability have hamstrung the application of the gene circuit method to larger networks or more broadly in development. Gene circuits have only been inferred for relatively small networks so far (Reinitz and Sharp 1995; Manu *et al.* 2009b; Cotterell and Sharpe 2010; May *et al.* 2013; Verd *et al.* 2018).

One approach to speeding up the inference procedure has been to explore different global optimization methods such as evolutionary algorithms (Kozlov and Samsonov 2009; Kozlov *et al.* 2012) and scatter search (Abdol *et al.* 2017). Alternative global optimization methods do not circumvent the problem of high computational cost since each cost function evaluation still involves the solution of coupled ODEs. Another important strategy for inferring gene circuits in a reasonable amount of time has been the development of parallel optimization algorithms such as parallel Lam simulated annealing (pLSA; Chu *et al.* 1999) and Differential Evolution Entirely Parallel (DEEP; Kozlov and Samsonov 2009; Kozlov *et al.* 2012), including attempts at reducing communication overhead (Jostins and Jaeger 2010; Lou and Reinitz 2016). Although parallel methods reduce the absolute amount of time required to infer a gene circuit of a given size, they nevertheless suffer from the scaling problem.

In this paper we present an alternative approach, FIGR (Fast Inference of Gene Regulation), for determining gene circuit parameters that is significantly more computationally efficient than global nonlinear optimization. Our algorithm exploits the observation that the inference of the connectivity of a given gene can be rephrased as a supervised learning problem: to find a hyperplane in state space that classifies observations into two groups, one where the gene is ON and the other where the gene is OFF. Our algorithm determines whether a gene is ON or OFF at a given observation point by computing the time derivative of concentrations in a numerically robust manner. It then performs classification using either logistic regression or support vector machines (SVM) to determine the equation of the switching hyperplane. The genetic interconnectivity can then be computed from the coefficients of the hyperplane equation in a straightforward manner. We have implemented the algorithm in MATLAB and tested its ability to recover the genetic interconnectivity of random GRNs of up to 50 genes from simulated data. The algorithm works as expected and recovers parameters accurately, provided that sufficient data are available. We also demonstrate the ability of our method to correctly infer the gap gene regulatory network of *Drosophila melanogaster* from empirical data. We observed a ∼ 600-fold speed up relative to simulated annealing on the gap gene problem.

The results are presented in two parts. In the first half of the results, we develop the algorithm and validate it on synthetic data. In the second half, we describe the inference of the *Drosophila* gap gene network from empirical data. We begin the first half by introducing gene circuit equations for cell-autonomous GRNs (Section Gene circuit models of GRNs). Next, we develop the FIGR algorithm (Section FIGR: Classification-based inference) for inferring gene circuits for cell-autonomous GRNs. This is followed by tests assessing the accuracy of FIGR in recovering known parameter values from synthetic data (Section Validation of FIGR on synthetic data). In the second half (Section Inference of the gap gene network from empirical data), we first adapt FIGR to spatially extended systems such as the *Drosophila* blastoderm (Section FIGR for a spatially extended system) and then compare FIGR-derived gap gene circuits to data and SA-derived gene circuits (Section Gap gene circuit inference and comparison with data and SA).

## Materials and Methods

### Validation of FIGR With synthetic data

#### Generation of synthetic data from random gene circuits:

Random gene circuits were generated and simulated to generate synthetic data as follows. The synthesis rates $R_{g}$ and degradation rates $\lambda_{g}$ were drawn uniformly from the interval [0.5,2] . We chose the genetic interconnectivity coefficients $T_{gf}$ and threshold $h_{g}$ such that the switching hyperplane passed through a random point $x^{\text{cen}}$ drawn uniformly from the bounding hypercube ($0<x_{g}^{\text{cen}}<\frac{R_{g}}{\lambda_{g}}$), and the normal to the switching hyperplane (${\hat{T}}_{g}$) was drawn uniformly from the unit *G*-sphere, where *G* is the number of genes in the GRN. We generated *N* trajectories starting at random initial position $x_{n}(t=0)$ drawn uniformly from the bounding hypercube by integrating the Glass equations without diffusion (Equation 3) using MATLAB’s ode45 solver. We stored the values of these functions $x_{ng}\left(t_{k}\right)$ at $N_{t}$ timepoints equally subdividing the interval of the simulation ([0,2]) to serve as synthetic data for both FIGR and SA.

#### Inference with FIGR:

Gene circuits were inferred using FIGR as described in Section FIGR: Classification-based inference. The user-defined options and parameters utilized in this study are provided in Table S1.

#### Inference with SA:

SA was carried out with gene circuit C code in serial largely as described previously (Manu *et al.* 2009b) save for a few modifications. The quality factor *λ* was set to 0.001 and the averaging parameters $\lambda_{u}$ and $\lambda_{v}$ were set to 200 and 1000 respectively. The stopping criterion was 0.001. The parameter controlling the search space of the regulatory parameters Λ was set to 0.1. The search space of $R_{g}$ and $\lambda_{g}$ were set to (0.4,2.1) . The Glass equations (Equation 3) were solved using a 4^th^ order Runge-Kutta solver. Since SA is a stochastic method, different optimization runs yield slightly different gene circuits. The inferences were carried out in 5 replicates, each starting for a random set of initial parameter values. For each synthetic dataset, several replicates having low RMS could be identified. The circuit with the lowest RMS was chosen for further analysis.

### Inference of gap gene circuits

Gap gene circuits were inferred from a publicly available whole-mount immunofluorescence dataset (Surkova *et al.* 2008) of the spatiotemporal expression of the segmentation genes. Data from the 50 min-long cleavage cycle 14 are staged into 8 time classes, T1–T8, spaced 6.25 min apart. We utilized integrated data (Manu *et al.* 2009b) obtained by subtracting background fluorescence from raw single-embryo data, aligning the spatial patterns of different embryos belonging to a time class, and averaging over several embryos in each time class. See Janssens *et al.* (Janssens *et al.* 2005) for details of how the data were processed.

The user-defined options and parameters utilized for fitting the gap gene circuit with FIGR are provided in Table S1. Wall clock time was measured with MATLAB’s tic/toc functions. SA was carried out with gene circuit C code as described previously (Manu *et al.* 2009b). Wall clock time was measured with C’s time function.

### Data availability

Figure S1 shows the fraction of genetic interconnectivity signs inferred correctly from synthetic data. Figure S2 shows the training error of SA-inferred gene circuits. Figure S3 shows the inference of $h_{g}$ and kinetic parameters from synthetic data. Figure S4 compares the spatiotemporal pattern of gap gene expression with the output of gene circuits inferred with FIGR and SA. Table S1 lists user-defined options and parameters utilized in FIGR code. File S1 describes an alternative method for determining kinetic parameters in FIGR. FIGR Source code is freely available at http://github.com/mlekkha/FIGR. Supplemental material available at figshare: https://doi.org/10.25387/g3.9249245.

## Results

### Gene circuit models of GRNs

We consider a GRN of *G* genes whose state at time *t* is defined by the concentrations of the gene products $x_{g}\left(t\right)$, g=1,2,…,G. We assume that the GRN functions cell autonomously, that is, the expression of the genes is independent of the state of other cells. Gene circuits (Reinitz and Sharp 1995) describe the time evolution of $x_{g}\left(t\right)$ according to *G* coupled ordinary differential equations,

$$\frac{dx_{g}}{dt}=R_{g}S\left(\overset{G}{\underset{f=1}{\sum }}T_{gf}x_{f}+h_{g}\right)-\lambda_{g}x_{g},$$ (1)

where $R_{g}$ is the maximum synthesis rate of product *g*. $T_{gf}$ are genetic interconnectivity coefficients describing the regulation of gene *g* by the product of gene *f*. Positive and negative values of $T_{gf}$ signify activation and repression of gene *g* by gene *f* respectively. The threshold $h_{g}$ determines the basal synthesis rate, and $\lambda_{g}$ is the degradation rate of product *g*. Nominally, all genes in the model also function as regulators, so that both *g* and *f* run over the range 1,2,3,…,G. Sometimes such gene networks include upstream regulators that are not themselves influenced by other gene products represented in the model. For example, in the *Drosophila* segmentation gene network, maternal proteins such as *bicoid* activate the zygotically expressed genes, but are not regulated by their targets (Akam 1987). An upstream regulator *g* can be represented by setting $T_{gf}=0$ for all *f*.

S(u) is the regulation-expression function, which determines the fraction of the maximum synthesis rate attained by the gene given the total regulatory input $u={\sum^{}}_{f=1}^{G}T_{gf}x_{f}+h_{g}$. S(u) is required to have a switch-like dependence on *u* and to take values between 0 and 1. If the total regulatory input has large positive values, u≫0, as a result of high activator concentrations, low repressor concentrations, or both, S(u)∼1 and the gene product is synthesized at the maximum rate $R_{g}$. If the total regulatory input has large negative values, u≪0, so that S(u)∼0, the gene product is not synthesized. One sigmoid function that satisfies these properties,

$$S\left(u\right)=\sigma \left(u\right)=\frac{1}{2}\left(\frac{u}{\sqrt{1+u^{2}}}+1\right),$$ (2)

has been utilized almost exclusively in previous studies (Reinitz and Sharp 1995; Jaeger *et al.* 2004a; Manu *et al.* 2009b; Kozlov *et al.* 2012). However, any function that satisfies these rather general properties is a valid regulation-expression function.

#### Glass networks:

In what follows, we show that one choice of the regulation-expression function permits a radical simplification of the gene circuit inference problem. If the regulation-expression function is chosen to be the Heaviside function,

$$S\left(u\right)=\Theta \left(u\right)=\{\begin{array}{ccc} 0 & \text{if} & u<0\\ 1 & \text{if} & u\geq 0. \end{array}$$

the resulting differential equations (Equation 1) are piece-wise linear and are referred to as Glass networks (Glass and Kauffman 1973; Edwards 2000; Glass and Pasternack 1978; Mestl *et al.* 1996).

Using the state vector $x=\left(x_{1},x_{2},\ldots ,x_{G}\right)$ to represent a point in the *G*-dimensional state space of the model and the vector $T_{g}$ to represent the gth row of the genetic interconnectivity matrix, the Glass equations may be written as

$$\frac{dx_{g}}{dt}=R_{g}\Theta (T_{g}\cdot x+h_{g})-\lambda_{g}x_{g},\;\;g=1,2,3,\ldots ,G.$$ (3)

The gene may said to be “ON” or “OFF” depending on whether the gene product is being synthesized or not respectively. Equation 3 implies that

$$\text{gene}\;g\;\text{is}\{\begin{array}{cc} \text{ON if} & T_{g}\cdot x+h_{g}>0\\ \text{OFF if} & T_{g}\cdot x+h_{g}<0. \end{array}$$ (4)

Thus the “gene *g* ON” and “gene *g* OFF” configurations are separated in state space by the hyperplane defined by the equation $T_{g}\cdot x+h_{g}=0$. We call this the *switching hyperplane*. $T_{g}$ is the normal to the switching hyperplane and $T_{g}\cdot x+h_{g}$ is the perpendicular distance of any point x to the hyperplane. Furthermore,

$$x_{g}\left(t\right)=\{\begin{array}{cc} x_{g}\left(0\right)e^{-\lambda_{g}t}+\frac{R_{g}}{\lambda_{g}}\left(1-e^{-\lambda_{g}t}\right) & \text{if ON for }t\geq 0\\ x_{g}\left(0\right)e^{-\lambda_{g}t} & \text{if OFF for }t\geq 0. \end{array}$$ (5)

**Figure 1:**
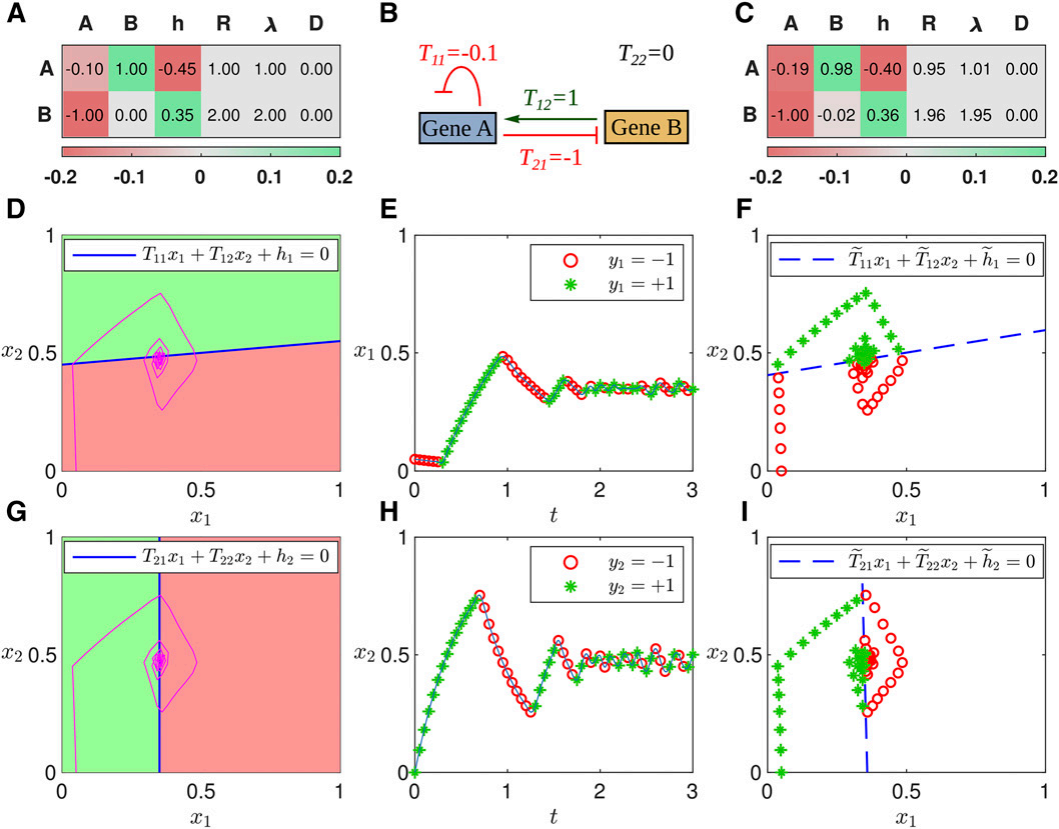
Classification-based inference of an example gene circuit. A. Theoretical parameter values are listed by row for each gene. The T matrix is shown in the first two columns, one column per regulator. Green (red) indicates activation (repression). B. Schematic of the theoretical gene circuit. C. Parameters inferred by FIGR. D,G. Trajectory in state space (purple) overlaid upon the Heaviside regulation-expression function. Green (red) is ON (OFF). The switching hyperplane is plotted as a blue line. Switching hyperplanes for genes A and B are showing in panels D and G respectively. E,H. Sampled gene expression trajectories and assignment of ON/OFF state for genes A (panel E) and B (panel H). Trajectories are numerical solutions of Equation 3. Detected ON or OFF state (Section Determining ON/OFF state) is indicated with green stars or red circles respectively. F,I. Switching hyperplane (dashed blue line) inferred using Logistic regression for genes A (panel F) and B (panel I). Sampled trajectories annotated with ON/OFF state are plotted in state space.

Equation 4 and Equation 5 imply that for Glass networks, the *regulatory parameters*, $T_{g}$ and $h_{g}$, and the *kinetic parameters*, $R_{g}$ and $\lambda_{g}$, are separable. The former determine the switching hyperplane, while the latter determine the trajectories on either side of the hyperplane. Figure 1D,G shows examples of the switching hyperplanes and trajectory of a two-gene gene circuit (Figure 1A,B) having a stable spiral equilibrium solution.

### FIGR: Classification-based inference

Let the expression of each gene be measured at $N_{t}$ time points $t_{e},e=1,\ldots ,N_{t}$, along trajectories starting from n=1,…,N initial conditions. The goal of GRN inference is to estimate the values of the gene circuit parameters ${\tilde{T}}_{gf}$, ${\tilde{h}}_{g}$, ${\tilde{R}}_{g}$, and ${\tilde{\lambda }}_{g}$ given the measurements $x_{ng}\left(t_{e}\right)$.

In FIGR, we exploit the separability of the regulatory ($T_{g}$, $h_{g}$ ) and kinetic ($R_{g}$, $\lambda_{g}$) parameters to break up the inference problem into two distinct tractable subproblems. For inferring the parameters of any given gene, we classify the data points into two classes—one in which the gene’s product is being synthesized (ON class) and the other in which the product is not being synthesized (OFF class). The regulatory parameters are inferred by determining the optimal G−1 dimensional hyperplane separating the two classes. The kinetic parameters can be inferred either by fitting the piece-wise linear Glass equations to estimates of the rate of change of gene product concentrations or by fitting Equation 5 to the gene product concentration time series.

#### Determining ON/OFF state:

We will assume that the gene product concentration, including initial concentration, is bounded by the maximum concentration determined by the synthesis and degradation rates, that is,

$$0\leq x_{g}<\frac{R_{g}}{\lambda_{g}},\;\;g=1,2,3,\ldots ,G.$$ (6)

Let

$$y_{g}\equiv \text{sgn}\left(T_{g}\cdot x+h_{g}\right)=\pm 1$$ (7)

represent the ON/OFF state of gene *g*. Then,

$$\frac{dx_{g}}{dt}=\{\begin{array}{ccc} R_{g}-\lambda_{g}x_{g}>0 & \text{if} & T_{g}\cdot x+h_{g}>0\\ -\lambda_{g}x_{g}\leq 0 & \text{if} & T_{g}\cdot x+h_{g}<0. \end{array}$$ (8)

This implies that the ON/OFF state of a gene can be determined by ascertaining the sign of the *velocity*, $v_{g}=\frac{dx_{g}}{dt}$ .

$$y_{g}\equiv \text{sgn}\left(T_{g}\cdot x+h_{g}\right)=\text{sgn}\frac{dx_{g}}{dt},\;\;g=1,2,3,\ldots ,G.$$ (9)

Gene expression data, such as those obtained from immunofluorescence or high-throughput sequencing, inevitably contain noise. If the gene expression level is close to its maximum ($x_{g}\approx R_{g}/\lambda_{g}$) or minimum level ($x_{g}\approx 0$), $\frac{dx_{g}}{dt}$ is theoretically close to zero, but noise causes $\text{sgn}\frac{dx_{g}}{dt}$ to fluctuate, which might be interpreted as spurious switching events. To avoid this problem, we identify a gene’s ON/OFF state as follows. If the gene expression level $x_{g}$ is increasing (decreasing) at a rate greater than a user-supplied *velocity threshold* $v_{g}^{c}$, then the gene is classified as ON (OFF). Otherwise, if the expression level is above (below) a user-supplied *expression threshold* $x_{g}^{c}$, then the gene is classified as ON (OFF). This can be summarized as

$$y_{g}=\{\begin{array}{cc} \text{sgn}\frac{dx_{g}}{dt} & |\frac{dx_{g}}{dt}|\geq v_{g}^{c}\\ \text{sgn}\left(x_{g}-x_{g}^{c}\right) & |\frac{dx_{g}}{dt}|<v_{g}^{c}. \end{array}$$ (10)

In our implementation of FIGR, cubic smoothing splines are fit to time series data and differentiated to estimate velocity. Figure 1E,H illustrates the determination of $y_{g}$ for an example two-gene network.

#### Determining regulatory parameters:

Within the Glass model, the ON/OFF state of a particular target gene *g*, whose index we shall omit from now on, is given by y=sgn(T⋅x+h). Suppose that gene product concentrations have been sampled *P* times, in time and in one or more conditions or cell types. The gene ON/OFF state $y_{p}$ is determined for each experimentally measured state vector $x_{p}$, p=1,2,3,…,P, according to the method described above (Section Determining ON/OFF state). Then, the regulatory parameters can be inferred by finding $\tilde{T}$ and $\tilde{h}$ such that

$$y_{p}=\text{sgn}\left(\tilde{T}\cdot x_{p}+\tilde{h}\right)$$ (11)

is satisfied for as many *p* as possible. Inferring the regulatory parameters therefore reduces to the problem of linear binary classification (Hastie *et al.* 2009).

There are many well known supervised learning algorithms for linear binary classification. We have used both support vector machines (SVM) and logistic regression. An SVM finds a hyperplane buffered by the biggest possible margin such that the number of points $x_{p}$ belonging to each class, “gene ON” or “gene OFF”, is maximized on opposite sides of the margin zone. This can be accomplished by minimizing the cost function

$$\chi \left(T,h,\lambda \right)=\lambda {\left|\left|T\right|\right|}^{2}+\overset{P}{\underset{p=1}{\sum }}L\left(y_{p},x_{p}\right),$$ (12)

where the first term is a regularization penalty that maximizes the margin. The second term is the hinge loss function, $L\left(y_{p},x_{p}\right)=\text{max}\left(0,1-y_{p}\left(T\cdot x_{p}+h\right)\right)$, which is non-zero only for points that transgress their class boundary, each such point contributing an amount proportional to its distance from the margin. The parameter *λ* is used to choose the relative weight of the penalty and loss terms. Two-class logistic regression models the posterior probability of the ON/OFF state of a point as a logit transformation of its distance from the switching hyperplane. The optimal switching hyperplane can be found by minimizing Equation 12 with a Binomial deviance loss function $L\left(y_{p},x_{p}\right)=\text{log}\left\{1+e^{-y_{p}\left(T\cdot x_{p}+h\right)}\right\}$. Figure 1C,F,I illustrate binary classification for an example two-gene network.

Minimization of Equation 12 is a convex optimization problem, which can be solved by quadratic programming or the Newton-Raphson method quite efficiently, even for large *G*. This is the key benefit of the separation of regulatory and kinetic parameters enabled by the Glass equations.

#### Determining kinetic parameters:

Having identified the ON/OFF state of a gene, $y_{p}$, for *P* measurements of its concentration, $x_{p}$, the Glass equations (Equation 3) can be rewritten as

$$v_{p}=\{\begin{array}{cc} R-\lambda x_{p} & \text{if }y_{p}=+1,\\ -\lambda x_{p} & \text{if }y_{p}=-1. \end{array}$$ (13)

The velocities $v=\frac{dx}{dt}$ are estimated by differentiating cubic smoothing splines fit to the time series data (Section Determining ON/OFF state). Thus, for any particular gene, Equation 13 takes the form of *P* equations that are linear in the two unknowns *R* and *λ*. This is an overdetermined linear system, so best estimates for *R* and *λ* can be extracted by least-squares linear regression. In practice, the error in the spline, and hence in *v*, is the largest when a gene is switching states. We therefore exclude a user-configurable number of time points nearest to switching events. This method is implemented as the **“slope”** method of FIGR. Alternatively, *R* and *λ* can also be determined by fitting Equation 5 to the concentration data (see File S1).

### Validation of FIGR on synthetic data

As a first test of FIGR, we tested its ability to recover known parameters from synthetic data. In each test, 100 randomly generated gene circuits (Section Validation of FIGR with synthetic data) were simulated using the Glass equations (Equation 3). For each gene circuit, *N* trajectories starting from random initial starting points were computed and sampled at $N_{t}$ time points to obtain synthetic time series data resulting in $N_{O}=N\times N_{t}$ observations per simulation. The quality of the inference depends not only on the effectiveness of the method but also on how well determined the inference problem is. A gene circuit of *G* genes has $N_{p}=G\left(G+3\right)$ parameters. If $N_{O}\gg N_{p}$, then the problem is well determined and the accuracy of the inference depends primarily on the effectiveness of the algorithm. On the other hand if $N_{O}∼N_{p}$ then there isn’t a sufficient amount of data to infer the parameters accurately irrespective of the effectiveness of the algorithm. We checked how effective FIGR is at inferring parameters of gene circuits of various sizes by exploring different combinations of the number of free parameters and the number of data points. With the exception of the 50-gene network, we also inferred the parameters with SA (Section Inference with SA) to serve as a point of reference. Inference of 100 random 20-gene networks took 5 days on 500 CPUs with SA, and hence it was impractical to infer 50-gene networks.

The inferred parameter values were compared to the known values by computing the discrepancies between them. From the viewpoint of correctly predicting a gene’s ON/OFF state, the accuracy of individual genetic interconnectivity coefficients $T_{gf}$ is less important than the accuracy with which the switching hyperplane has been inferred. Accordingly, we judged the accuracy of the genetic interconnectivity matrix by computing the magnitude of the vector difference between the unit normals of the theoretical ($T_{g}$) and inferred (${\tilde{T}}_{g}$) hyperplanes, $\delta_{T}=\left|\left|{\tilde{T}}_{g}-T_{g}\right|\right|$. When the angle between the unit normals is small, $\delta_{T}$ gives the angle between them. $\delta_{T}=\sqrt{2}$ implies that the inferred hyperplane is orthogonal to the theoretical one, and $\delta_{T}=2$ is the maximum value possible, implying that the two normals are in exactly opposite directions and the assignment of ON/OFF state has been reversed. We also computed the null distribution of $\delta_{T}$ which results from choosing the inferred unit normal at random uniformly on the unit *G*-sphere. The discrepancies in the other parameters were computed as $\delta_{h}=|{\tilde{h}}_{g}-h_{g}|$, $\delta_{R}=|{\tilde{R}}_{g}-R_{g}|$, and $\delta_{\lambda }=|{\tilde{\lambda }}_{g}-\lambda_{g}|$, where ${\tilde{h}}_{g}$, ${\tilde{R}}_{g}$, and ${\tilde{\lambda }}_{g}$ are inferred parameter values.

**Figure 2:**
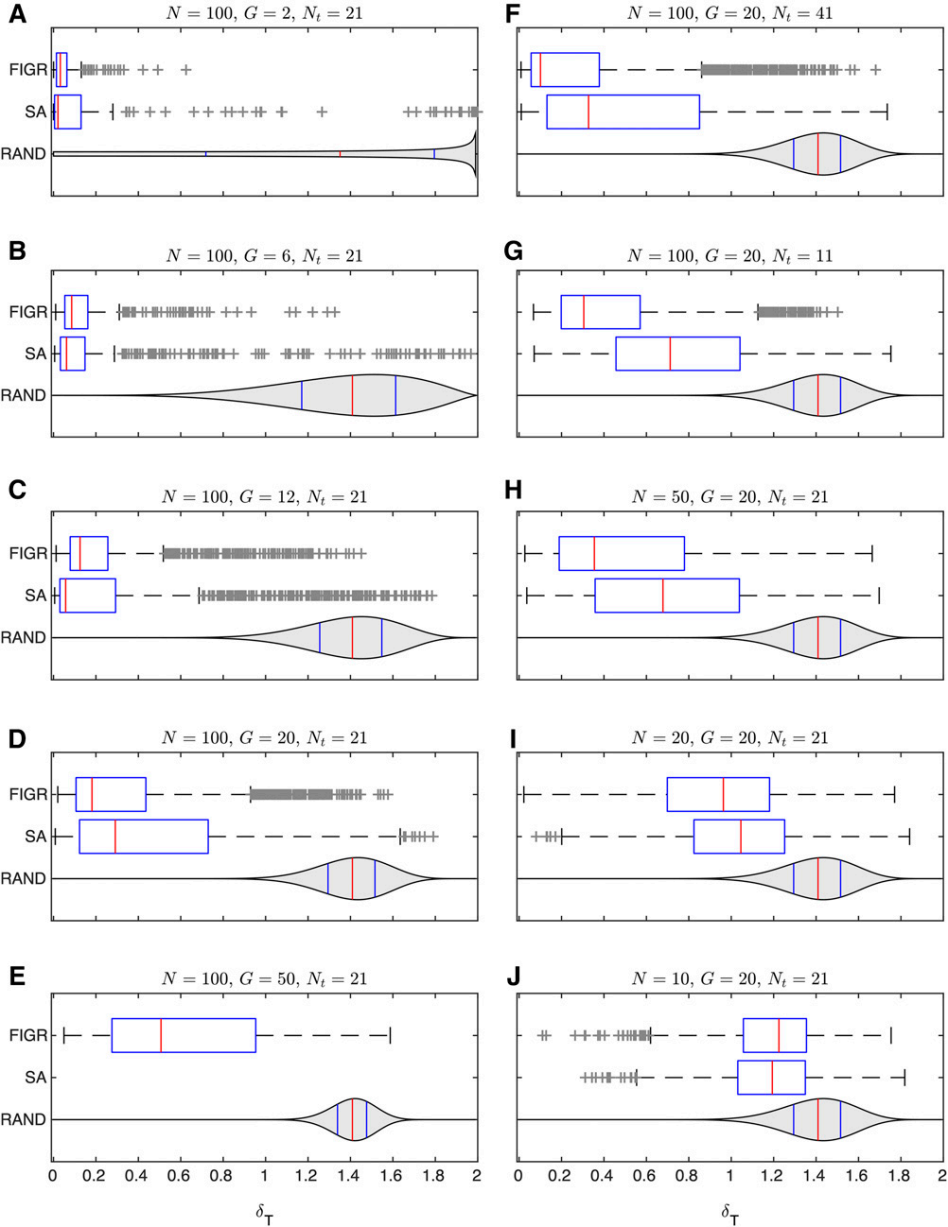
Inference of genetic interconnectivity coefficients from synthetic data. The distribution of the discrepancy between inferred and theoretical switching hyperplanes, $\delta_{T}=\left|\left|{\tilde{T}}_{g}-T_{g}\right|\right|$, in inferring 100 random gene circuits with FIGR or SA is shown as boxplots. ${\tilde{T}}_{g}$ and $T_{g}$ are unit normals to the inferred and theoretical hyperplanes. Note that each boxplot is constructed from 100G discrepancies since each random parameter set contains *G* switching hyperplanes, where *G* is the number of genes. The box lines are the first quartile, median, and the third quartile. The whiskers extend to the most extreme values lying within 1.5 times the interquartile range, and any datapoints outside the whiskers are shown as crosses. The blue violin plot (“RAND”) shows the null discrepancy distribution that would be obtained, $P_{0}\left(\delta_{T}\right)\propto \delta_{T}^{G-2}{\left(1-\frac{\delta_{T}^{2}}{4}\right)}^{\frac{G-3}{2}}$, if ${\tilde{T}}_{g}$ were not inferred but instead picked randomly from a uniform distribution over the surface of the *G*-sphere. The width of the violin plot is proportional to $P_{0}\left(\delta_{T}\right)$, and blue and red vertical lines indicate quartiles and median respectively. A–E. Number of trajectories N=100 and number of timepoints $N_{t}=21$. The number of genes *G* was varied between 2 and 50 for FIGR and 2 and 20 for SA, since SA was impractical for G=50. F,G. Number of trajectories N=100 and number of genes G=20. The number of timepoints $N_{t}$ was varied between 41 (panel F) and 11 (panel G). H–J. Number of genes G=20 and number of timepoints $N_{t}=21$. The number of trajectories N was varied between 10 and 50.

In the first set of simulations, we simulated networks of size ranging from two to fifty genes (Figure 2A–E) with N=100 trajectories sampled at $N_{t}=21$ time points. The switching hyperplane is inferred with high accuracy ($\delta_{T}<0.1$ or 6° angle to the theoretical normal) for the vast majority of two-gene random gene circuits, showing that FIGR is capable of recovering the true values of the parameters if a sufficient amount of data are available. As the size of the gene circuit, and consequently the number of free parameters, increases, the accuracy declines. However the inference is still fairly accurate for 20-gene networks since 75% of the inferred hyperplanes have $\delta_{T}<0.5$ or less than a 30° angle to the theoretical hyperplane. Inferring the signs of the genetic interconnectivity coefficients $T_{gf}$, that is, whether a regulator activates or represses a target, is an important goal in gene circuit analysis. FIGR achieves 90% accuracy in inferring the signs of $T_{gf}$ for 20-gene networks (Fig. S2). For 50-gene networks, the accuracy of quantitative inference is quite low and most inferred hyperplanes have $\delta_{T}>0.5$ or larger than a 30° angle to the theoretical hyperplane. This is not entirely surprising since a 50-gene network has 2,650 free parameters, while the model is being inferred from only 2,100 observations. Although 50-gene networks are inferred with poor accuracy, the $\delta_{T}$ distribution is significantly better than the null distribution, suggesting that the inferred parameters still contain information about GRN structure. In fact, the signs of 82% interconnectivity coefficients are inferred correctly (Fig. S1) suggesting that FIGR still works reasonably well at a qualitative level in a highly underdetermined problem.

Hyperplanes inferred with SA also show a trend of decreasing accuracy with increasing network size, suggesting that declining accuracy is a result of progressive reduction in the determinacy of the problem rather than an intrinsic inability of FIGR to infer larger networks. In fact, in nearly all cases, SA’s inferences were more variable than FIGR, and were slightly less accurate than FIGR on the 20-gene problem. The relatively lower accuracy of SA is not a result of poor fitting since the RMS of most of the random gene circuit fits is less than 0.04 (∼4% error; Fig. S2).

In the second set of simulations, we simulated random 20-gene networks, but varied the number of sampled time points $N_{t}$ (Figure 2F,G) or the number of trajectories *N* (Figure 2H–J). Increasing or decreasing the number of time points to 41 or 11 respectively had a minimal effect on the quality of the inference of the switching hyperplanes by FIGR or SA (compare to Figure 2D). This suggested that 11 time points were sufficient to reliably estimate the genetic interconnection coefficients. In contrast, decreasing the number of trajectories progressively reduced accuracy and both FIGR and SA inferences were indistinguishable from the null distribution when only 10 trajectories were sampled. Once again, this is not surprising since the 460 free parameters of a 20-gene network are being inferred from only 210 observations. These results imply that while increasing temporal resolution beyond a certain point provides diminishing gains in accuracy, the number of trajectories or conditions the trajectories are sampled from is a crucial parameter influencing the quality of the inference.

The inference of $h_{g}$ (Fig. S3) was quite accurate and behaved like the inference of the switching hyperplanes. Increasing gene network size or reducing the amount of data tended to reduce accuracy, although the effects were less pronounced than what was observed while inferring switching hyperplanes. The kinetic parameters were also inferred accurately by FIGR (Fig. S3). The accuracy of both $R_{g}$ and $\lambda_{g}$ increased with increasing number of time points but did not depend on the number of genes or trajectories. This can be understood as a consequence of the separation of regulatory and kinetic parameters in Glass gene circuits—the inference of the kinetic parameters occurs independently for each gene and depends only on the sampling frequency.

Although the inference is quite accurate, the discrepancies are not zero for most gene circuits and can be fairly large for a small number of gene circuits, even in the 2-gene case. This results from constraints imposed by the intrinsic dynamics of gene circuits and finite sample size. For instance, trajectories move away from the switching hyperplane for autoactivating genes. In this case, the initial conditions act as support vectors for the inferred hyperplane, which then strongly depends upon the random sample of starting points. Another situation that arises is that of a hyperplane that divides the bounding hypercube into ON and OFF regions in a lopsided manner. Since initial points are sampled uniformly, this results in too few sampled points in the vicinity of the hyperplane and poor inference. Given that FIGR was at least as accurate as SA, these failure modes are not specific to the inference methodology but likely represent fundamental limitations of inferring differential equations models. These considerations are also valid when inferring GRNs from empirical data. Such insights and their implications for parameter identifiability will be described elsewhere. Notwithstanding these constraints, our analysis demonstrates that FIGR is capable of inferring parameters quantitatively when provided with a sufficient number of data points and qualitatively (signs of $T_{gf}$) even when the problem is underdetermined as in the 50-gene case.

### Inference of the gap gene network from empirical data

We tested the efficacy of FIGR on empirical data by inferring a gene circuit for the gap gene network acting during *Drosophila* segmentation. The gap gene network is one of the best characterized developmental GRNs at both the experimental and theoretical levels, and therefore serves as a benchmark for gene circuit inference. We provide a very brief summary of the segmentation system here; a more complete description may be found in reviews by Akam (Akam 1987) or Jaeger (Jaeger 2011). The segmentation proteins pattern the anteroposterior axis during the first three hours of embryogenesis. During this period, the embryo is a syncitium, so that nuclei lack cell membranes and undergo 13 mitotic divisions, termed cleavages. After the tenth cleavage cycle, the majority of the nuclei migrate to the periphery of the embryo and are arranged in a monolayer, forming a syncitial blastoderm. Cellular membranes form by invagination of the plasma membrane in the latter half of cleavage cycle 14, at the end of which the embryo undergoes gastrulation.

Near the end of cleavage cycle 14, the segmentation genes are expressed in spatially resolved patterns that specify the position of each cell to an accuracy of one cell diameter. Segmentation gene expression is initiated by shallow protein gradients formed by the translation of localized mRNAs, such as *bicoid* (*bcd*) and *caudal* (*cad*), deposited in the oocyte by the mother. These maternal protein gradients regulate the gap genes, which commence mRNA expression in cleavage cycle 10–12 and are expressed in broad domains ∼ 20 nuclei wide during cycle 14. The gap genes in turn regulate the pair-rule and segment-polarity genes that provide the molecular prepattern for the subsequent segmentation of the embryo. All the maternal and gap proteins are known to act as transcription factors that regulate each others’ expression in a complex GRN that has been modeled extensively (Reinitz *et al.* 1995; Jaeger *et al.* 2004a,b, 2007; Gursky *et al.* 2008; Vakulenko *et al.* 2009; Manu *et al.* 2009a,b; Gursky *et al.* 2011; Ashyraliyev *et al.* 2009; Wotton *et al.* 2015; Verd *et al.* 2018).

#### Gene circuit equations for a spatially extended system:

The gap gene circuit describes the time evolution of the concentrations of the gap proteins in a one-dimensional row of *N* nuclei lying along the anteroposterior axis of the embryo (Manu *et al.* 2009b) during cleavage cycle 14. The lack of cell membranes in the syncitium implies that proteins can diffuse between nuclei (cells) and Equation 1 is modified to incorporate discretized Fickian diffusion and anteroposterior position, so that

$$\begin{array}{l} \frac{dx_{ng}}{dt}=R_{g}\sigma \left(\overset{G}{\underset{f=1}{\sum }}T_{gf}x_{nf}+h_{g}\right)\\ +D_{g}\left(x_{n-1,g}+x_{n+1,g}-2x_{n,g}\right)\\ -\lambda_{g}x_{ng}. \end{array}$$ (14)

Here $x_{ng}\left(t\right)$ is the expression level of protein *g* in nucleus *n* at time *t*, $D_{g}$ is the diffusion constant for protein *g*, and σ(u) is the sigmoid regulation-expression function (Equation 2). Zero-flux boundary conditions are used at the ends of the modeled region, while initial conditions are given by the empirical data from cycle 13.

#### FIGR for a spatially extended system:

The determination of ON/OFF state and inference of regulatory parameters $T_{g}$ and $h_{g}$ is carried out as described above in Sections and. However, inferring gene circuits in spatially extended systems requires two modifications to the algorithm. First, the diffusion constants $D_{g}$ must be inferred in addition to $R_{g}$ and $\lambda_{g}$. Second, for gene circuits with diffusion, the correspondence between velocity $v_{g}$ and ON/OFF state $y_{g}$ (Equation 9) will be violated for a few nuclei lying adjacent to nuclei in which the gene is ON. Such nuclei would have positive velocity despite the gene being OFF because of diffusion of the protein from adjacent “ON nuclei”. Although this effect is limited to a few nuclei, it would nevertheless result in slightly inaccurate parameter estimates. For this reason, once the regulatory and kinetic parameters have been inferred, they are refined by local optimization.

#### Inference of kinetic parameters including $D_{g}$:

We exploit so-called kink solutions to the gene circuit equations (Vakulenko *et al.* 2009) to estimate the kinetic parameters. Let gene *g* be ON in an anteroposterior domain [l,r] so that there is net diffusion out of the domain into surrounding OFF nuclei. Then the balance of synthesis, degradation, and diffusion will establish a stable gradient,

$$x_{ng}\left(t\right)=\{\begin{array}{cc} \frac{R_{g}}{2\lambda_{g}}e^{-\gamma_{g}\left(n-r\right)}, & n>r,\\ \frac{R_{g}}{2\lambda_{g}}e^{-\gamma_{g}\left(l-n\right)}, & n<l, \end{array}$$ (15)

outside the domain. Here, $\gamma_{g}=\sqrt{\frac{\lambda_{g}}{D_{g}}}$ is the characteristic length scale of the gradient at steady state.

In order to infer the kinetic parameters, we first determine the time class in which a gene is expressed at the highest level, since the spatial gradient would best approximate steady state at that time point. Next, we identify gene-expression domains as local maxima in the spatial pattern in that time class. For genes having multiple expression domains, such as *hb* and *gt*, the domain with the highest expression is chosen. The gene is expressed at half maximum at the domain borders (Equation 15). Therefore, border positions (*l* or *r*) are determined as nuclei where the expression is half of the domain peak. $R_{g}$, $\lambda_{g}$, and $D_{g}$ are then determined by fitting Equation 15 to the observed expression data for n>r and n<l using MATLAB’s lsqnonlin function. This is implemented as the **“kink”** method of FIGR.

### Refinement of parameters

The parameter values estimated using classification and fitting to kink solutions serve as a starting point for an unconstrained local search using the Nelder-Mead algorithm implemented in MATLAB’s fminsearch function. The cost function is $\chi^{2}=\sum_{ngt}{\left({\tilde{x}}_{ng}\left(t\right)-x_{ng}\left(t\right)\right)}^{2}$, where ${\tilde{x}}_{ng}\left(t\right)$ are data and $x_{ng}\left(t\right)$ are solutions of Equation 14.

#### Gap gene circuit inference and comparison with data and SA:

**Figure 3:**
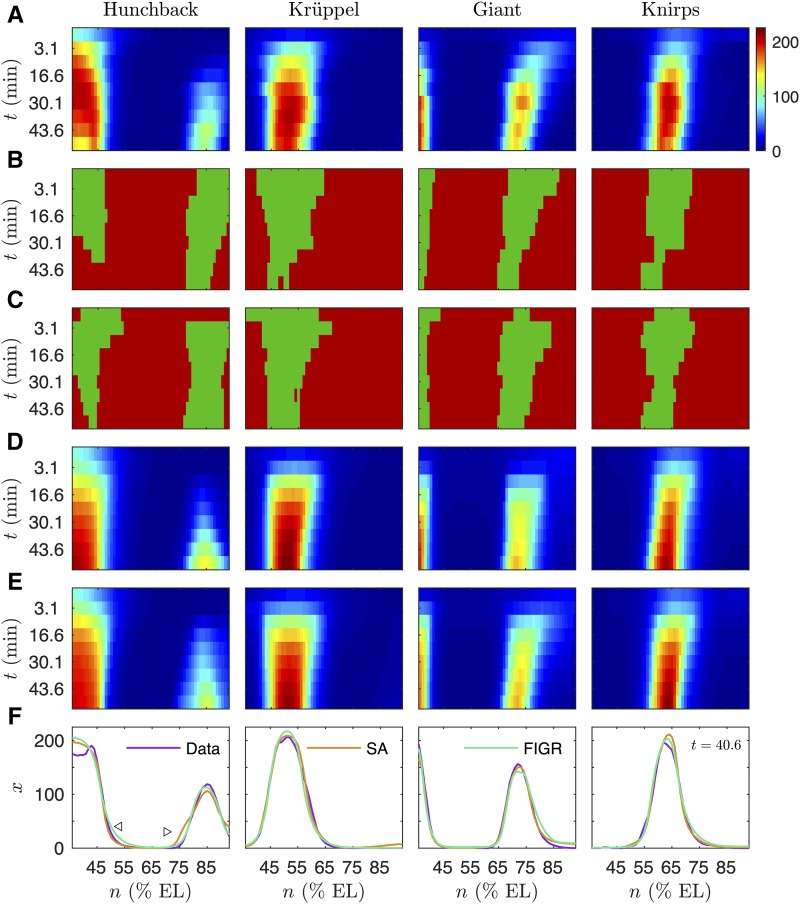
Classification-based inference of the *Drosophila* gap gene network. Horizontal axes indicate anteroposterior position. Vertical axes represent time elapsed since the thirteenth nuclear division in the downward direction. A. Integrated spatiotemporal gap gene expression data $x_{ng}\left(t_{k}\right)$, where k=1,…,9, used to infer the gene circuit. Data at the first time point are from cycle 13 and serve as initial conditions for the model. The rest of the time points correspond to eight time classes in cycle 14 separated by 6.25 min. Hot (cold) colors indicate high (low) expression levels. B. Classification of ON/OFF state. Green (red) colors indicate ON (OFF) states $y_{ng}\left(t_{k}\right)$ determined from velocities $v_{ng}\left(t_{k}\right)$. C. Prediction of ON/OFF state ${\tilde{y}}_{ng}\left(t_{k}\right)$ using parameter values inferred with FIGR. D. Gene circuit output using parameters obtained by FIGR and then refined using local search (RMS = 13.29). E. Gene circuit output using parameters inferred by SA (RMS = 11.03). F. Comparison of the spatial patterns of gap gene expression (blue), output of gene circuit inferred by SA (red; SA), and output of gene circuit inferred by FIGR (green) at t=40.6 min (time class T7). Slightly shallow posterior border of the anterior Hb domain in FIGR output is indicated with ◁. Slight ectopic bump in the anterior border of the posterior Hb domain in SA output is indicated with ▷.

We inferred a gene circuit for the gap genes *hunchback* (*hb*), *Krüppel* (*Kr*), *giant* (*gt*), and *knirps* (*kni*) using both FIGR (Section FIGR for a spatially extended system) and SA (Section Inference of gap gene circuits). The model includes the upstream regulators *Bicoid* (*Bcd*), *Caudal* (*Cad*), and *Tailless* (*Tll*), so that G=7. Our implementation matched previous gap gene models (Manu *et al.* 2009b) so that the upstream proteins regulated the trunk gap genes but not vice versa. The time-dependent concentrations of Bcd, Cad, and Tll were determined by linear interpolation of the expression data. The gene circuit models the protein expression of these genes between 35–92% egg length during cleavage cycle 14 (Section Inference of gap gene circuits). The dataset is comprised of immunofluorescence protein concentration measurements at the resolution of individual nuclei at eight timepoints during cleavage cycle 14 (Figure 3A).

In the first step of FIGR, the ON/OFF state of each gene is determined by estimating the velocity $v_{g}$ (Section Determining ON/OFF state). Figure 3B shows that the assignment of ON/OFF states by FIGR correctly recapitulates the extent and dynamics of the gene expression domains. In the next step, the regulatory parameters ${\tilde{T}}_{g}$ and ${\tilde{h}}_{g}$ were inferred by supervised classification (Section Determining regulatory parameters). Predictions of the ON/OFF state from the inferred parameter values, ${\tilde{y}}_{g}=\text{sgn}\left({\tilde{T}}_{x}\cdot x_{g}+{\tilde{h}}_{g}\right)$ (Figure 3C), are in good agreement with the empirical ON/OFF classification and the gene expression domains, suggesting correct inference of the regulatory parameters. In the third step, the kinetic parameters were estimated by fitting the kink equations to gap gene domain borders (Section Inference of kinetic parameters including $D_{g}$.). In the presence of diffusion, the correspondence between velocity $\frac{dx_{ng}}{dt}$ and the ON/OFF state of the gene will be violated for nuclei abutting gene expression domains due to diffusion of protein from ON regions into OFF regions. The inferred parameters likely deviate slightly from optimal values and were therefore refined using local search (Section Refinement of parameters.) in the fourth step of the algorithm. During refinement, the solutions were computed using gene circuit equations that represent synthesis with the sigmoid regulation-expression function (Equation 14) instead of the Heaviside function. Therefore the final model obtained did not rely on the assumption of binary switch-like regulation, which was utilized only during binary classification while obtaining the initial guesses of parameter values.

Gene circuit simulation with refined parameters produces output that agrees very well with the data and the output of gene circuits inferred using SA (Figure 3D,E), and has an RMS ($\chi /\sqrt{n}$) of 13.29. Careful comparison of the data and the outputs of the FIGR- and SA-derived gene circuits shows that there are only minor discrepancies between model output and data (Figure 3F and Fig. S4), some shared between FIGR and SA output and a few unique to each method. Both methods underpredict Hb, Kr, and Gt expression in time class T1 (t=3.1 min), a discrepancy that is resolved at later time points (Fig. S4). In FIGR output the posterior border of the abdominal Gt domain retracts a bit earlier, in time class T2, than in data, another discrepancy that is resolved at later time points (Fig. S4). SA output has an ectopic bump in the anterior border of the posterior Hb domain while FIGR produces a slightly shallower posterior border of the anterior Hb domain (Figure 3F). Despite these minor discrepancies, FIGR output agrees well with data overall, having the same border and peak positions, and reproduces the dynamic anterior shifts in gap gene domains during cleavage cycle 14 (Fig. S4).

**Figure 4:**
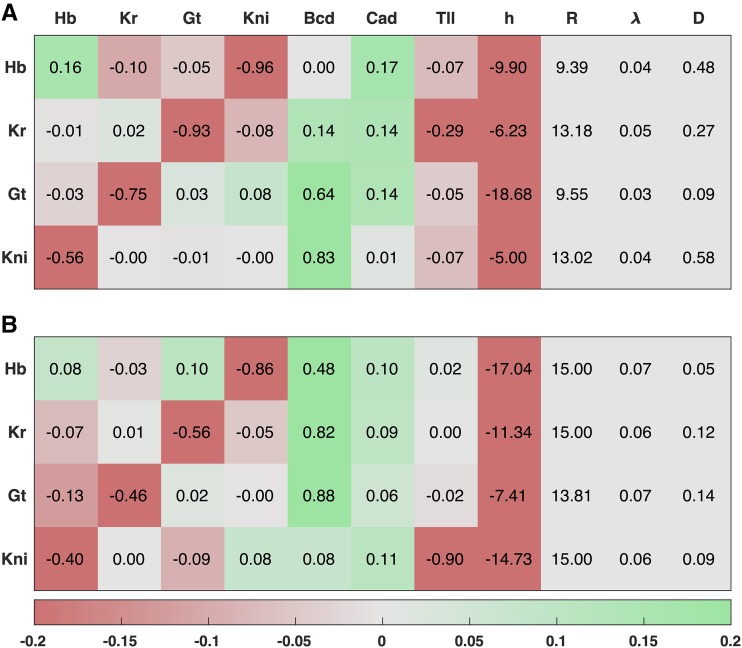
Comparison of gap gene parameters inferred by FIGR and SA. The values of ${\tilde{T}}_{g}$, ${\tilde{h}}_{g}$, ${\tilde{R}}_{g}$, ${\tilde{\lambda }}_{g}$, and ${\tilde{D}}_{g}$ are listed one gene per row. The T matrix is shown in the first seven columns, one column per regulator. Green (red) indicates activation (repression). Since $T_{g}$ and $h_{g}$ can only be determined up to a constant factor, they have been normalized ($\left\|\left({\tilde{T}}_{g1},\ldots ,{\tilde{T}}_{gG},{\tilde{h}}_{g}\right)\right\|=1$) to allow comparison between FIGR and SA. A. FIGR after refinement. B. SA.

The inferred gap gene network (Figure 4) is in good agreement with experimental evidence (Jaeger 2011), the network inferred by SA, and previous analyses (Manu *et al.* 2009b; Jaeger *et al.* 2004a). One of the organizing principles underlying the resolution of gap gene domains, termed “alternating cushions”, is that genes with non-overlapping domains, Kr and Gt or Hb and Kni, strongly repress each other (Kraut and Levine 1991). The genetic interconnection coefficients of Kr/Gt and Hb/Kni inferred by FIGR have negative values of high magnitude, which is consistent with the alternating cushions mechanism. Another key feature reproduced by FIGR output is the movement of posterior gap gene domains to the anterior during cycle 14 (Jaeger *et al.* 2004b). These shifts have been understood to occur in a cell-autonomous manner (Jaeger *et al.* 2004b) due to asymmetric repression between gap genes in reverse order of adjacent gap gene domains, Kr ⊢ Kni ⊢ Gt (Manu *et al.* 2009a). The FIGR gene circuit encapsulates this mechanism since 1) genes expressed in adjacent domains repress each other weakly or not at all and 2) Gt represses Kni but not vice versa and Kni represses Kr but Kr does not repress Kni.

The activation of the gap genes inferred by FIGR is also consistent with experimental evidence. The gap genes are activated by Bcd and Cad (Driever and Nüsslein-Volhard 1989; Rivera-Pomar *et al.* 1995; Hoch *et al.* 1991; Eldon and Pirrotta 1991; Schulz and Tautz 1995). Hb autoactivates strongly, in agreement with experimental evidence (Margolis *et al.* 1995; Simpson-Brose *et al.* 1994; Hülskamp *et al.* 1994). Although there isn’t clear evidence that the other gap genes autoactivate (Jaeger 2011), the FIGR GRN, like previously inferred ones (Jaeger *et al.* 2004a; Manu *et al.* 2009b; Kozlov *et al.* 2012), features weak autoactivation by Kr and Gt. Two main differences are observed between the FIGR- and SA-inferred gene circuits. First, whereas Hb is strongly activated by Bcd in the SA gene circuit, it is only weakly activated in the FIGR gene circuit. However in other studies using both wildtype (Jaeger *et al.* 2004a) and wildtype/*Kr*^−^ (Kozlov *et al.* 2012) data, SA infers Bcd → Hb interactions ranging from repression to strong activation. A second difference is that whereas Tll activates Hb in the SA circuit, it represses Hb in the FIGR gene circuit. Experimental evidence about whether Tll activates or represses Hb in the posterior is inconclusive (Jaeger 2011). In summary, the agreement between FIGR output and data are the result of genetic mechanisms that have been substantiated by previous experimental and modeling analyses.

With compiled MATLAB code, FIGR with refinement takes 48 sec compared to 29,067 sec taken by SA (∼600-fold speed up) to achieve the same RMS. In summary, FIGR infers the gap gene network accurately but at a fraction of the computational expense.

## Discussion

Gene circuits (Reinitz and Sharp 1995; Jaeger *et al.* 2004a; Manu *et al.* 2009b) provide many unique advantages for inferring and modeling developmental GRNs. The differential equations are biologically realistic in representing gene regulation as a nonlinear switch-like function of TF concentrations. Gene circuits not only infer the topology of the network but the directionality (causality), sign (activation/repression), and strength of regulatory interconnections. Most importantly, gene circuits are not limited to inference but are capable of accurately simulating and predicting gene expression patterns. Finally, the use of differential equations allows gene circuits to compute transient solutions, an important factor in simulating development since fate determination can occur before equilibrium is reached (Manu *et al.* 2009a; Simcox and Sang 1983). Despite the promise held by gene circuits, their application, as of other data-driven differential equation models, has been limited to smaller networks so far. Analysis of larger networks is limited to correlative approaches (Margolin *et al.* 2006; Segal *et al.* 2003) that neither infer causality nor simulate or predict the time evolution of GRN state.

A major challenge in broader application of gene circuits is the high computational expense of inferring the free parameters from time series data. Currently, the approach for inferring parameter values (Chu *et al.* 1999; Reinitz and Sharp 1995; Kozlov *et al.* 2012; Abdol *et al.* 2017) is to solve (“integrate”) the ODEs to obtain trajectories, compare with experimental trajectories, and refine parameters using global optimization techniques such as SA. This procedure is slow and expensive because it requires performing multidimensional optimization on a complicated cost function $\chi^{2}\left(\left\{T,h,R,\lambda \right\}\right)$ with many local minima and each function evaluation involves solving a system of ODEs. Moreover, the computational complexity grows rapidly ($O\left(G^{3}\right)$) so that global optimization approaches for gene circuits scale poorly with G.

In contrast, FIGR directly attempts to fit the differential equations, which describe how the velocities $v_{g}$ depend upon the concentrations $x_{g}$. $T_{g}$ and $h_{g}$ are determined using binary classification (support vector machines or logistic regression). Both of these algorithms reduce to quadratic programming, and thence to convex optimization. Subsequently, $R_{g}$ and $\lambda_{g}$ can be determined by linear regression against velocities or non-linear regression against concentrations using the piece-wise analytical solutions of the ODEs, which are even simpler optimization problems. Each inference can be completed in a fraction of a second on a consumer-grade computer, even with interpreted MATLAB code. We found that in the gap gene circuit, the values of the regulatory parameters $T_{g}$ and $h_{g}$ thus inferred were consistent with those obtained by SA, and ODEs solved with the inferred parameters produced gap gene domains correctly positioned in space and time. Furthermore, the parameter values, which were inferred under the assumption of a Heaviside regulation-expression function, served as a good starting point for equations with a sigmoid regulation-expression function. Gene circuits with a good RMS could be obtained with an “off-the-shelf” Nelder-Mead simplex method built into MATLAB. This refinement takes under a minute in serial compared to the eight hours taken by SA. Finally, logistic regression has a computational complexity of O(G), where *G* is the number of features (genes), which implies that FIGR has a complexity of $O\left(G^{2}\right)$ when inferring *G* genes. Consequently, FIGR scales with problem size much better than global nonlinear optimization techniques.

The computational efficiency of FIGR does not come at the expense of accuracy. In testing the recovery of known parameters from synthetic data (Figure 2, S1, and S3), we found that FIGR and SA had comparable accuracy for smaller gene circuits, while FIGR had slightly higher accuracy than SA for 20-gene networks. We speculate that the lower accuracy of SA results from “sloppiness” (Gutenkunst *et al.* 2007; Ashyraliyev *et al.* 2008; Kozlov *et al.* 2012)—insensitivity of model output to certain genetic interconnectivity coefficients. If a certain parameter gives similar solutions over some interval, then SA can infer any value in the interval rather than the true value. This insensitivity can result from the compensatory and redundant roles many parameters play in the model (Ashyraliyev *et al.* 2008; Kozlov *et al.* 2012). For example, a high expression level can be achieved by having higher activating genetic interconnection coefficients, by having higher synthesis rates, or by having lower degradation rates. The higher accuracy of FIGR could perhaps be attributed to the separation of the regulatory and kinetic parameters, which limits the opportunities available for redundant parameters to produce similar solutions. It is not possible to decide which method was more accurate in inferring the gap gene circuit because the “true” values are not known. However, there are hints that FIGR suffers less from the compensation problem in the gap gene case as well. Using SA, it is common to get fits where a gap gene is either activated by Bcd and has low basal synthesis ($h_{g}<0$) or a gap gene is repressed by Bcd but has high basal synthesis ($h_{g}>0$). The former is biologically accurate, while the latter is not. For this reason, in the SA optimizations performed here and in previous work (Jaeger *et al.* 2004a; Manu *et al.* 2009b; Gursky *et al.* 2011; Kozlov *et al.* 2012; Perkins *et al.* 2006), $h_{g}$ was constrained to be negative. No such constraint was used in FIGR, and it infers low basal synthesis for all the gap genes autonomously.

In representing synthesis as a binary ON/OFF choice, Glass equations (Equation 3) are similar to Boolean or logical models, which have been applied to a broad range of developmental GRNs (Theiffry *et al.* 1993; Sánchez and Thieffry 2001; Davidson *et al.* 2002b; Thieffry and Sánchez 2003; Collombet *et al.* 2017; Bonzanni *et al.* 2013). Given this similarity between Boolean models and Glass equations, FIGR should be readily applicable to a large class of GRN modeling problems. Moreover, Glass equations relax the assumption made in logical models—that genes are expressed at a small number of discrete levels—to allow expression at any arbitrary level. This makes Glass models more general than Boolean models and capable of simulating transient dynamics during development. The accurate inference of the gap gene network using FIGR (Section Gap gene circuit inference and comparison with data and SA) demonstrates that the assumption of discrete ON/OFF synthesis may also be relaxed—binary classification provided estimates of parameters which were close enough to the optimal values that further refinement by a local search resulted in good fits.

The separation of the inference procedure into regulatory and kinetic components bears a superficial similarity to the multi-step approach of Perkins *et al.* (Perkins *et al.* 2006) for inferring gap gene circuits. In the first step of their approach, the diffusion and decay parameters are inferred by assuming that the domains where each gap gene is synthesized have a quadrilateral shape in space-time. In the second step, the regulatory parameters are inferred by fitting a sigmoid synthesis function to the quadrilateral production domains inferred in the first step, followed by a third step of refining all parameters by solving the coupled differential equations. In contrast to the method of Perkins *et al.* our method does not assume any particular shape for the production domains and does not require manual analysis to define the domains. The production domains result naturally and automatically from our classification procedure (Figure 3B). The lack of a strong assumption about the shape of expression domains means that FIGR should be applicable to genes having any production domain shape, and with 2D or 3D spatial data. The second point of divergence is that we infer the regulatory parameters using binary classification instead of fitting the sigmoid synthesis function to the production domains. As a consequence, FIGR is faster than the method of Perkins *et al.*

Although in our tests FIGR was shown to be at least as effective as, and much faster than, SA, it does have a few limitations. First, in order to estimate the velocity and determine ON/OFF state reliably, FIGR requires that the data be sampled sufficiently frequently in time. Roughly speaking, during each time period in which a gene is in a particular state (ON or OFF), its product concentration would have to be sampled at least three times in order to ascertain the velocity and state. FIGR therefore would not be suitable for datasets that have been sampled sparsely in time. Methods reliant on solving the ODEs will, in contrast, attempt to fit the trajectories to a sparsely sampled dataset, even if the actual inference achieved is poor (Figure 2J). FIGR was successful in inferring the gap gene circuit even though the gap gene dataset (Figure 3A) has only 8 time points because most nuclei don’t change state and a few nuclei at gene expression borders change state once, allowing sufficient sampling for a reliable detection of the state.

In spatially extended systems, the sign of the velocity, which can be estimated empirically, and the unobserved gene ON/OFF state are not strictly equivalent due to diffusion of the gene product out of the region of synthesis. In the gap gene system, we found that the sign of the velocity nevertheless corresponded quite well to gene state (Figure 3B); well enough, in fact, to provide a starting point for a local search that yielded good fits (Figure 3D). The success of this approximation is likely due to the fact that gap gene patterning is largely cell-autonomous and diffusion plays the limited role of averaging gene expression in space (Manu *et al.* 2009a; Jaeger *et al.* 2004b; Gursky *et al.* 2004). In spatial systems where patterning is largely driven by diffusion (Kondo and Miura 2010), FIGR’s decomposition of regulatory and kinetic parameters might be less successful, and require a greater reliance on the refinement of parameters by solving the differential equations. Successfully extending FIGR to spatially extended systems besides the gap gene network may require the recognition of situations where patterning is driven by diffusion. The simplest empirical means of assessing the importance of diffusion is to compare mRNA and protein expression patterns. A large overlap between the two would imply that diffusion is less important and that parameter estimates obtained through binary classification would be close to optimal. If, on the other hand, protein expression domains extend much farther than the mRNA expression domains, it would imply that the initial estimates are not close to optimal values and more aggressive refinement is likely needed. If mRNA expression data are not available or difficult to obtain, it might be possible to estimate parameters under slow or fast diffusion scenarios by constraining the ON domains to be larger or smaller respectively during binary classification. This could potentially be achieved by adjusting the velocity and expression thresholds $v_{g}^{c}$ and $x_{g}^{c}$ prior to binary classification in FIGR.

Besides the problem of computational efficiency, the broader application of gene circuits, and indeed all nonlinear differential equation models, is limited by a lack of understanding of parameter identifiability. Most commonly, *a posteriori* confidence intervals for the parameter estimates are computed (Ashyraliyev *et al.* 2008). Such calculations are based on the strong assumption that the solution is linear in the parameters and that the measurement errors are normally distributed. *a posteriori* parameter identifiability analysis also does not provide any hints to improve experimental design for achieving better identifiability in future studies.

Although we have not directly addressed the problem here, we anticipate that conceiving of and visualizing gene circuit inference as a classification problem will lead to insights into parameter identifiability. For example, it is evident from state space plots (Figure 1F,I) that sampling gene expression trajectories closer to the true switching hyperplane of the gene will lead to less “wiggle room” for the inferred hyperplane and result in more accurate parameter inference. This implies that datasets that measure gene expression near steady state, for example in differentiated cell types, are unlikely to lead to accurate parameter inference irrespective of the number of data points sampled or the precision of the experiment. Sampling transient trajectories densely when genes are turning ON or OFF is the best strategy for accurate parameter inference. Another less obvious implication is that it is easier to infer the regulation of negatively autoregulated genes than positively autoregulated ones. Trajectories move toward or away from the switching hyperplane for negatively or positively autoregulated genes respectively, making it more likely that sampled data points will lie near the hyperplane in the former. This analysis will be reported elsewhere.

In summary, we have exploited features of the mathematical structure of gene circuits to break a difficult optimization problem into a series of two, much simpler, optimization problems. We have demonstrated that FIGR is effective on synthetic as well as experimental data from a biologically realistic GRN. We have validated the inferred gap gene model by comparing its parameters against models inferred with SA as well as comparing its output against experimental data. The improvement in computational efficiency and scalability should allow the inference of much larger GRNs than was possible previously.
